# Rotatum of light

**DOI:** 10.1126/sciadv.adr9092

**Published:** 2025-04-11

**Authors:** Ahmed H. Dorrah, Alfonso Palmieri, Lisa Li, Federico Capasso

**Affiliations:** ^1^ Harvard John A. Paulson School of Engineering and Applied Sciences, Harvard University, Cambridge, MA 02138, USA.; ^2^ Department of Applied Physics and Science Education, Eindhoven University of Technology, Eindhoven 5612 AP, Netherlands.

## Abstract

Vortices are ubiquitous in nature and can be observed in fluids, condensed matter, and even in the formation of galaxies. Light, too, can evolve like a vortex. Optical vortex beams are exploited in light-matter interaction, free space communications, and imaging. Here, we introduce optical rotatum, a behavior of light in which an optical vortex beam experiences a quadratic chirp in its orbital angular momentum along the optical path. We show that such an adiabatic deformation of topology is associated with the accumulation of a Gouy phase factor, which, in turn, perturbs the propagation constant (spatial frequency) of the beam. The spatial structure of optical rotatum follows a logarithmic spiral—a signature that is commonly seen in the pattern formation of seashells and galaxies. Our work expands the previous literature on structured light, offers new modalities for light-matter interaction, communications, and sensing, and hints at analogous effects in condensed matter physics and Bose-Einstein condensates.

## Teaser

A class of optical vortices with chirped orbital angular momentum is generated for use in light-matter interaction and sensing.

## INTRODUCTION

Vortex flow is a signature of many systems in nature and is often seen in turbulent fluids, smoke rings, tornados, electric and magnetic currents, and even the formation of galaxies (*1*). Electromagnetic radiation, including light, can also evolve like a vortex both in space (*2*, *3*) and time (*4*–*7*). Optical vortex beams are typically characterized by an azimuthal phase dependence of *e*^*i*ℓϕ^, where ℓ denotes the slope of the phase (*8*). Such a profile carries an on-axis phase singularity, which forces the Poynting vector to skew off-axis. This nonzero transverse component of the Poynting vector, in turn, creates orbital angular momentum (OAM) of ℓℏ per photon (*9*, *10*). Besides their rich physics, vortex beams have enabled new degrees of freedom for light-matter interaction (*11*–*13*) and have been used in free space communications (*14*, *15*), remote sensing (*16*–*18*), imaging (*19*, *20*), quantum information processing (*21*–*23*), among many other applications (*24*, *25*). A variety of tools have been used to generate optical vortex beams, including digital holography (*26*, *27*), metasurfaces (*28*–*30*), spiral (*31*), and geometric phase plates (*32*).

OAM is a conserved quantity under free space propagation and is associated with a quantized topological charge ℓ. Thus, OAM cannot be freely modified (*33*–*35*). Nevertheless, several complex patterns of vortex beams have been reported thanks to an abundance of advanced wavefront shaping tools. For instance, the vorticity of light can now be locally modulated along the optical path (*36*–*42*). In this case, it is understood that the OAM density may vary locally (at the center of the beam) while keeping the global OAM conserved at each plane along the propagation direction (*37*, *43*). Vortex beams of this kind have been used in refractometry by mapping light’s rotation to the unknown refractive index (*17*), high-capacity free space communications by transmitting different symbols to different receivers located along the optical path (*44*), and in robust information transfer by matching the structured beam to the spatially varying turbulence profile of the medium (*45*).

Different techniques have been used to spatially modulate the topological charge of optical vortex beams with propagation. A common strategy relies on interfering multiple copropagating OAM modes with different ℓ values and propagation constants such that their spatial beating produces an envelope that changes its OAM, locally, with propagation (*36*–*38*). This approach has demonstrated vortex beams that change their topological charge from one integer value to another following a step-like transition. In this case, the vortex beam carries integer values of ℓ or superpositions thereof. Continuous evolution of OAM, spanning fractional (*46*) and integer ℓ values, has also been demonstrated by transmitting light through spiral slits (*41*, *42*, *47*). The linear growth or decay of OAM in space can be controlled by engineering the geometry of the spiral. The temporal analog of this behavior is referred to as self-torque of light (τ), where light’s vorticity changes linearly as a function of time giving rise to a nonzero first-order derivative of OAM (L_*z*_), such that τ = *d*L_*z*_/*dt* (*5*, *6*). Light of this kind provides an extraordinary tool for laser-matter manipulation on attosecond time and nanometer spatial scales. It also poses many new questions, for example, can light change its self- torque with propagation (i.e., *d*^2^L_*z*_/*dt*^2^ ≠ 0)? Although higher-order derivatives (second, third...etc.) of OAM have been observed and studied in classical mechanics (*48*), referred to as jerk or rotatum, their electromagnetic analog has not been introduced in the literature to date despite their rich physical dynamics and potential applications. Electromagnetic rotatum brings a new optical forces toolkit, which can be exploited in sorting colloids in three-dimensional (3D) (*13*, *49*), or as topological drivers for the electronic transitions in emerging quantum materials (*50*), and laser-plasma interaction (*51*).

In this work, we experimentally demonstrate a class of vortex beams, which carry optical rotatum. Optical rotatum describes vortex beams whose ℓ value experiences a quadratic chirp along the optical path, giving rise to a nonzero second-order derivative of OAM (i.e., *d*^2^L_*z*_/*dz*^2^ ≠ 0)—a quantity that has not been observed in electrodynamic systems to date. The mechanism relies on introducing an azimuthally varying gradient in the spatial frequency (*k*-vector) of the beam. Hence, in analogy to vortex beams, which carry an azimuthal phase gradient, here, each point on the azimuth is characterized by a slightly different *k*-vector. Upon propagation, different points along the azimuthal direction of the wavefront will accumulate different phase delays, causing the phasefront to acquire a singularity on axis while experiencing a continuous deformation in its helical twist. By judiciously designing this azimuthal *k*-gradient (*dk*_*z*_/dϕ), it is possible to generate vortex beams whose OAM can locally follow any polynomial dependence (i.e., linear, quadratic, or cubic...etc.) along the optical path. Notably, such an adiabatic evolution in OAM gives rise to a Gouy phase factor, which perturbs the propagation constant of the beam and that can be tailored by design. Besides their rich dynamics, light beams of this kind can be used as optical rulers for precise depth sensing and metrology and can also find application in the efficient sorting of colloids in 3D, to name but a few. Our work expands on the current literature of structured light generation, hints at similar observations in many other physical systems in nature, and can be applied beyond optics, for example, in ultrasonic (*52*) and electron beams (*53*).

### Concept

**Fig. 1.**
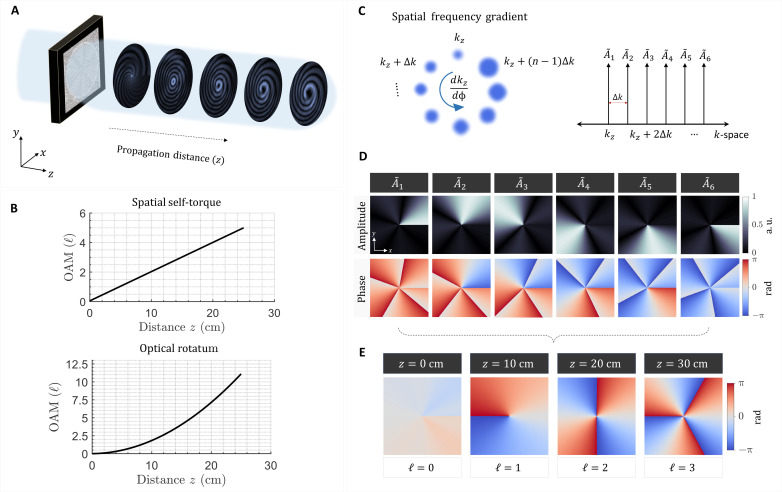
Rotatum of light. (**A**) A phase mask converts a plane wave into a vortex beam whose OAM can grow (or decay) following a quadratic dependence along the direction of propagation. (**B**) The OAM, signified by ℓ, can follow any arbitrary *z*-dependent profile, which can be linear, quadratic, or cubic. The linear and quadratic evolution of OAM gives rise to the spatial self-torque (top) and rotatum of light (bottom), respectively. (**C**) The mechanism relies on introducing an azimuthal gradient in the spatial frequency of the beam, *dk*_*z*_/dϕ (left). This can be realized by creating a spatial frequency comb in the *k*_*z*_ domain. Each comb tooth is weighted by a complex coefficient, ${\tilde{A}}_{n}\left(\phi \right)$ (right). (**D**) Amplitude and phase profiles of the coefficients ${\tilde{A}}_{n}\left(\phi \right)$, designed in this case to produce a vortex beam with linearly evolving OAM. Each coefficient, ${\tilde{A}}_{n}\left(\phi \right)$, is associated with a different spatial frequency, *k*_*z*,*n*_. a.u., arbitrary units. (**E**) Upon propagation, different components of the beam, associated with different *k*-vectors, and weighted by ${\tilde{A}}_{n}\left(\phi \right)$, will interfere forming an envelope with unity amplitude and *z*-dependent phase profile, which adiabatically deforms its helical twist along the optical path via spatial beating.

We seek a phase mask that converts an incident plane wave into a vortex beam that changes its OAM in a parabolic manner along the optical path, as illustrated in Fig. 1A. The OAM (ℓ) shall evolve continuously following a linear, quadratic, or even cubic *z* dependence. Here, we focus on linear and quadratic OAM evolution as depicted in Fig. 1B. The former can be interpreted as a spatial self-torque of light, whereas the latter is its rotatum. Tailoring the evolution of OAM to follow a parabolic *z* dependence requires the wavefront to change its helical twist continuously as light propagates. In other words, any two points in the azimuthal direction should accumulate slightly different phase shifts upon propagation. To achieve this, we introduce an azimuthal gradient in the spatial frequency of the beam. Consequently, the propagation constant will also vary, point-by-point, in the azimuthal direction. As the beam propagates, it acquires a helical wavefront, which continuously deforms its twist following any predetermined profile. Figure 1C illustrates this concept: A discrete set of monochromatic wave sources with different propagation constants are arranged in a ring formation. Each of these sources (or modes) has a different *k*_*z*,*n*_ vector, with equal separation in *k*_*z*_-space, and is weighted by different complex coefficients, ${\tilde{A}}_{n}\left(\phi \right)$. Here, *k*_*z*_ denotes the longitudinal component of the wave vector and *n* is the index of each azimuthal mode.

The coefficients ${\tilde{A}}_{n}\left(\phi \right)$ depend on the target OAM profile. For example, let the desired vortex beam evolve following *e*^*i*ℓ(*z*)ϕ^ dependence (where ℓ is now a function of *z*) over a finite distance of *L*. To obtain ${\tilde{A}}_{n}\left(\phi \right)$, we solve the following Fourier integral

$${\tilde{A}}_{n}\left(\phi \right)=\frac{1}{L}\int_{0}^{L}e^{i\ell \left(z\right)\phi }e^{-i\frac{2\pi n}{L}z}\mathrm{dz}$$ (1)

Equation 1 yields a discrete set of 2D phase and amplitude profiles whose superposition constructs the target function *e*^*i*ℓ(*z*)ϕ^. This is specifically true if the *k*-vector separation associated with two consecutive modes, ${\tilde{A}}_{n}\left(\phi \right)$ and ${\tilde{A}}_{n-1}$, is $k_{z,n}-k_{z,n-1}=2\pi /L$.

The first six coefficients ${\tilde{A}}_{n}\left(\phi \right)$ for a wavefront that evolves as ∼*e*^*i*10zϕ^ are plotted in Fig. 1D. Those coefficients are sufficient to engineer the underlying interference mechanism. When added together and propagated, the resulting envelope will acquire a quasi-uniform phasefront at *z* = 0 and helical phasefronts with ℓ = 1 and ℓ = 2, at *z* = 10 cm and *z* = 20 cm, respectively, as shown in Fig. 1E. Hence, by substituting any target OAM profile in the Fourier integral of Eq. 1, one can find the coefficients ${\tilde{A}}_{n}\left(\phi \right)$ of each azimuthal mode. The next step is to determine the profile of our propagating modes (i.e., the spatial carriers), which provide the ∼*e*^*ik*_*z*.*n*_*z*^ dependence. Bessel beams are ideal candidates for this purpose given their nondiffracting and self-healing behavior (*54*). Traditionally constructed by axicons or plane waves along a cone, Bessel beams have a spatial frequency (propagation constant), which can be precisely tuned by changing their cone angle—a feature that perfectly fits our approach. To formulate the propagating fields in terms of higher-order Bessel modes, however, we need to decompose the coefficients ${\tilde{A}}_{n}\left(\phi \right)$ onto the *e*^*i*ℓϕ^ basis, such that

$${\tilde{A}}_{n,m}=\frac{1}{2\pi }\int_{0}^{2\pi }{\tilde{A}}_{n}\left(\phi \right)e^{-\mathrm{im\phi}}d\phi$$ (2)

Therefore, the profile of our proposed beams is mathematically expressed as (*37*)

$$\psi \left(\rho ,\phi ,z,t\right)=\sum_{n=-N}^{n=N}\sum_{m=-M}^{m=M}{\tilde{A}}_{n,m}J_{m}\left(k_{\rho }^{n,m}\rho \right)e^{\mathrm{im}\phi }e^{{\mathrm{ik}}_{z}^{n,m_{z}}}e^{-i\omega t}$$ (3)

Here, *J*_*m*_ denotes the Bessel function of the first kind of order *m* whereas *k*_ρ_ and *k*_*z*_ are the transverse and longitudinal wave numbers, respectively, and the term *e*^−iωt^ denotes the harmonic time dependence. The summation consists of 2*N*+1 sets of Bessel ensembles. Each set contains 2*M*+1 Bessel beams of the same order *m*. By solving this equation at *z* = 0, one can obtain the 2D profile of the field distribution, which, upon propagation, will acquire the desired helical phase profile *e*^*i*ℓ(*z*)ϕ^.

## RESULTS

**Fig. 2.**
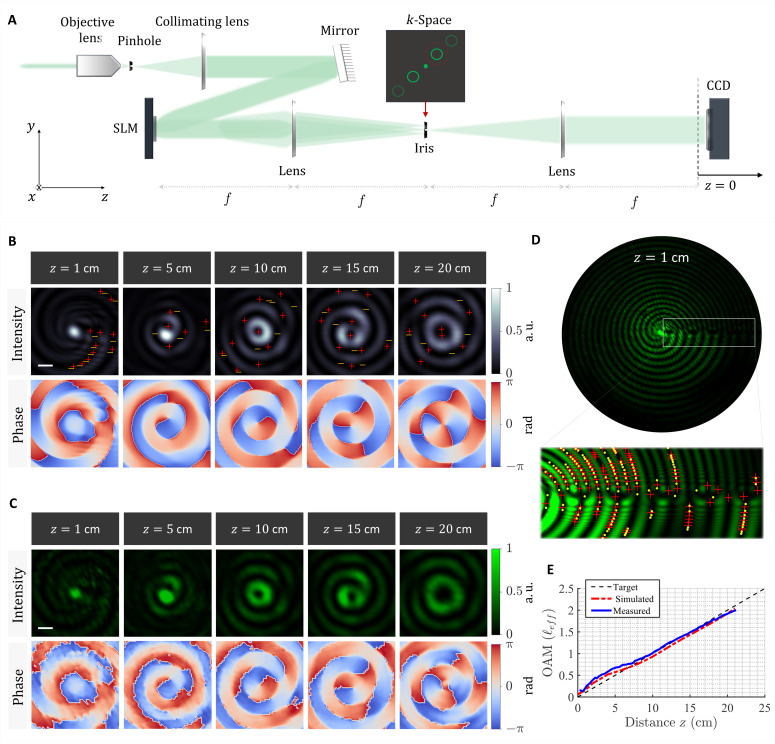
Generation of a vortex beam with spatial self-torque. (**A**) Experimental setup: An expanded and collimated laser beam is incident on a reflective phase-only SLM with the desired hologram. The reflected beam is filtered and imaged onto a CCD using a 4-*f* lens system. The CCD is mounted on a translation stage to capture the transverse profile of the beam at different *z* planes. (**B**) Simulated 2D profiles of the intensity and phase of a vortex beam in which the OAM increases linearly along the optical path. The red and yellow markers denote phase singularities with positive and negative handedness. Scale bar is 100 μm. (**C**) Measured 2D intensity and reconstructed phase profiles of the beam in (B). (**D**) The initial beam profile (at *z* = 1 cm) exhibits a horizontal line of darkness and bifurcation. The inset depicts the underlying chain of phase singularities associated with this dislocation line. (**E**) Measured and simulated evolution of OAM (effective charge, ℓ_eff_) as a function of propagation distance.

Substituting *z* = 0 in Eq. 3 provides the complex field profile that shall be implemented on the wavefront shaping tool of choice, for example, metasurfaces or spatial light modulators (SLMs). To generate our target field profiles, a standard holography setup composed of a phase-only reflective SLM and 4-*f* imaging system was used as depicted in Fig. 2A and described more fully in Materials and Methods. In the following, we demonstrate the experimental generation of optical vortex beams in which the OAM varies linearly and quadratically along the optical path. We also discuss the underlying physical dynamics associated with such evolution.

### Linearly growing vortex beams

We start with a scenario in which a vortex beam experiences a linear chirp in its OAM as it propagates. To achieve this, the vortex beam shall acquire a helical phasefront that is a linear function of *z*, following ∼*e*^*i*ℓ(*z*)ϕ^ dependence. The chirp rate of ℓ as well as its sign can be designed at will to create a vortex beam with linearly increasing (or decreasing) OAM. A linear monotonic variation in OAM as a function of *z* is the spatial self-torque, as described in Introduction. To be specific, consider a beam in which the target helical phase is given by ∼*e*^*i*10ϕz^. The coefficient 10 is a normalization constant, which defines the linear chirp rate of the azimuthal phase—i.e., slope of the linear OAM evolution—along the optical path. This particular choice of chirp rate allows the beam to increase its OAM (ℓ) by an integer value every 10 cm. It could have been set to other values as well. To realize the desired vortex beam, we evaluate ${\tilde{A}}_{n}\left(\phi \right)$ by solving Eq. 1 and then we obtain the initial field distribution from Eq. 3. By doing so, we find that the coefficients ${\tilde{A}}_{n}\left(\phi \right)$ are given by the closed form expression

$${\tilde{A}}_{n}\left(\phi \right)=-\frac{e^{2\phi i-\left(\frac{4\pi n}{5}\right)i-i}}{5\phi -2\pi n}$$ (4)

The six most considerable terms of ${\tilde{A}}_{n}\left(\phi \right)$ are the same as the ones depicted in Fig. 1D, exhibiting six amplitude masks with different azimuthal orientations. Choosing a different chirp rate for OAM would change the number of these azimuthal masks, producing finer or coarser sectors, which translates to modifying the azimuthal gradient in the *k*-vector. This is the case because each ${\tilde{A}}_{n}\left(\phi \right)$ will be multiplied by a Bessel beam with different *k*_*z*_. By substituting ${\tilde{A}}_{n}\left(\phi \right)$ in Eq. 2 and evaluating the expression in Eq. 3 at *z* = 0, the 2D field distribution that shall be encoded on the SLM is obtained. A plane wave incident on the SLM would thus be transformed to a vortex beam whose topological charge follows the linear dependence ℓ = 10*z*, chosen by design. The accuracy of constructing such a profile, however, relies on including a large number of terms (*M*) in the Fourier series of Eq. 3, which can be computationally demanding. To accelerate the calculation of the phase masks, the following approximation was adopted: ${\tilde{A}}_{n,m}={\tilde{A}}_{n}\left(\phi \right)$ and *M* = 0. This approximation slightly perturbs Eq. 3 as an exact solution to the wave equation (see fig. S1) but allows us to explore a large parameter space for our beams with closed form expression of ${\tilde{A}}_{n}\left(\phi \right)$.

Figure 2B depicts the simulated 2D intensity and phase profiles of the resulting beam at different propagation distances. The profiles were obtained using the Kirchhoff-Fresnel diffraction integral. Notice how the local wavefront slowly evolves to a helical profile as the beam propagates. The positive and negative phase singularities in the vicinity of the beam’s center are denoted by the red and yellow markers. They signify local charges of opposite handedness. The spatial movement of these singularities play a role in modifying the local topological charge at the center of the beam from ℓ = 0 at *z* = 1 cm to ℓ = 1 at *z* = 10 cm and ℓ = 2 at *z* = 20 cm. The measured intensity and phase profiles are shown in Fig. 2C and are in very good agreement with the simulated ones in Fig. 2B. The phase has been reconstructed from the intensity measurements using the single-beam multiple-intensity reconstruction (SBMIR) technique (*55*) described in section S8. The evolution of the beam’s phase and intensity profiles is captured in movie S1.

A closer look at the initial beam profile (at *z* = 1 cm), depicted in Fig. 2D, reveals two key features of this vortex beam: first, a region of bifurcation or dislocation (*56*) due to the mismatch in the spatial frequency along the azimuthal direction. This discontinuity arises precisely where the fast and slow spatial oscillations of the Bessel beam merge. See, for example, Fig. 1C, the region that lies between the sections associated with *k*_*z*_ and *k*_*z*_ + (*n* − 1)Δ*k*. Second, a chain of phase discontinuities (i.e., off-center isolated singularities) of alternating polarity. This chain of isolated singularities is a universal feature of OAM beams with fractional topological charge (*46*, *56*, *57*).

To confirm the linear growth of OAM, we calculate the effective topological charge (ℓ_eff_) of the beam (*9*, *58*, *59*). In essence, ℓ_eff_ is proportional to the ratio between the total OAM and energy of a given field distribution. It is expressed as ℓ_eff_ = ωL_*z*_/W and provides a quantitative measure of the OAM per photon (*58*, *59*). The derivation for calculating effective topological charge can be found in the second subsection of Materials and Methods. Figure 2E depicts the simulated and experimentally evaluated ℓ_eff_ as a function of propagation distance in comparison with the target linear profile. Here, ℓ_eff_ has been evaluated locally by encircling a finite region of 200 μm around the beam’s center. From this result, it can be inferred that the OAM is locally chirped along the optical path following the desired linear profile. This effect is different from previous demonstrations (*37*, *43*) in which ℓ_eff_ was allowed to acquire integer values or weighted superpositions thereof. Notably, linear chirp in ℓ_eff_ occurs only locally at the center of the beam. The global OAM integrated over the entire cross section of the beam is always conserved, aided by the movement of the phase discontinuities (off-center isolated singularities) across the beam’s peripheral (*37*, *43*). We illustrate this further in figs. S2 and S3. This mechanism is general and can allow other types of OAM evolution, which can be nonmonotonic or even parabolic as we will show. In the next example, we demonstrate another set of vortex beams whose charge follows a nonmonotonic evolution (i.e., growth and decay) and we point to an underlying Gouy-type phase factor that accompanies such transition.

### Nonmonotonic linear OAM evolution

**Fig. 3.**
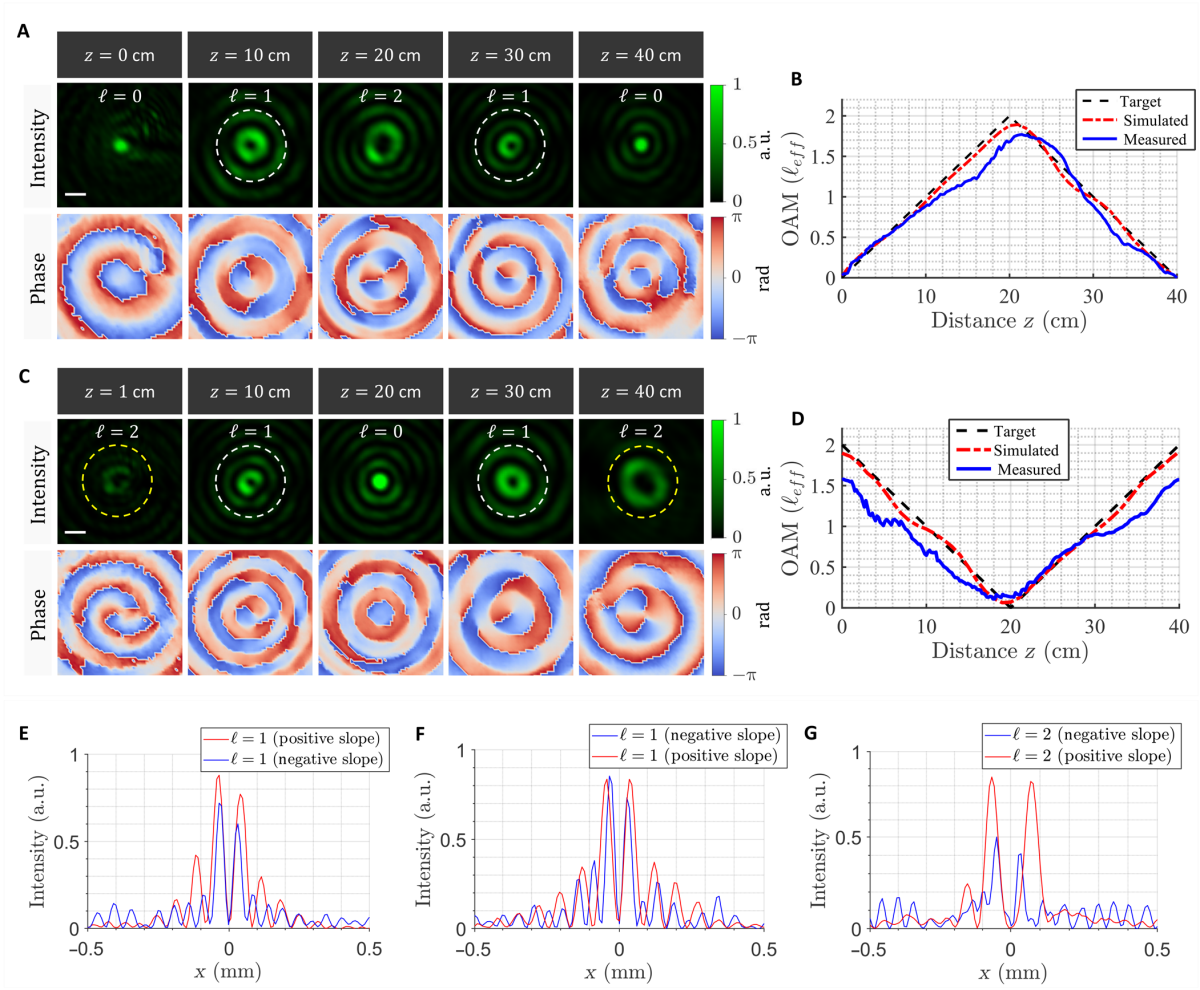
Optical vortex beams with nonmonotonically varying topological charge. (**A**) Measured transverse profiles (intensity and phase) of a vortex beam whose OAM locally grows then decays along the optical path. The beam acquires a helical phase, increasing its OAM adiabatically from ℓ = 0 to ℓ = 2, in a linear manner over a range of 20 cm. Afterward, the local OAM decreases continuously from ℓ = 2 to ℓ = 0 as the beam propagates for longer distance. Scale bar, 100 μm. (**B**) Measured and simulated spatial evolution of OAM (effective charge, ℓ_eff_) in comparison with the target design for the optical vortex beam in (A). (**C**) Measured 2D intensity and phase profiles of an OAM beam whose vorticity decays and then grows with propagation. The beam starts with a local charge of ℓ = 2 and then slowly unwinds its helicity to ℓ = 0 before it acquires the same helical phase (ℓ = 2) again, albeit with different size. (**D**) Measured and simulated evolution of the corresponding charge (ℓ_eff_) as a function of *z*. Vertical 1D cuts of the transverse profiles in (A) and (C), marked by the dashed circles, are plotted in (**E** to **G**), respectively. These cuts suggest that the beam’s size is perturbed (i.e., it experiences a *k*-shift) even when the topological charge is the same. The change in size depends on whether ℓ_eff_ increases or decreases with propagation.

Although it seems counterintuitive, a vortex beam that reverses its torque can be realized using our approach. This can be done by superimposing two sets of vortex beams, where the first experiences a linear growth in OAM (as shown before) whereas the second experiences a decay. Vortex beams with decaying OAM can be realized by setting the target phase profile to *e*^*i*(ℓ_o_−ℓ(*z*))ϕ^, where ℓ_o_ is the initial charge of the beam and −ℓ(*z*) signifies the negative slope of charge evolution. To demonstrate this further, we present two scenarios in which a vortex beam experiences a linear growth and then decay in its OAM and vice versa. Figure 3A depicts the measured 2D intensity and phase profiles for the first case: A pencil-like beam with a localized spot and uniform phasefront at the center slowly acquires a helical phase, evolving to a vortex beam with varying strength and diameter, before it rewinds its helicity and transforms back to a pencil beam. Notably, although the beam carries the same charge, ℓ = 1, at *z* = 10 cm and *z* = 30 cm, the beam’s diameter is slightly perturbed at these positions. This effect is predicted from the simulated profiles based on Kirchhoff’s propagation (see fig. S3) and will be explained shortly. The corresponding evolution of OAM (local charge), considering a 200-μm aperture around the beam’s center, is shown in Fig. 3B, confirming the linear dependence on *z*. The dynamical evolution of the same beam is further captured in movie S2.

To contrast this picture, we generated another vortex beam whose local OAM decays then grows linearly with propagation. Figure 3C and movie S3 show the measured transverse intensity and phase profiles for this case. Here, a vortex beam with ℓ = 2 continuously unwinds its helical wavefront until it becomes locally uniform at *z* = 20 cm before it acquires a helical phase again, increasing its charge to ℓ = 2. The corresponding evolution in OAM is plotted in Fig. 3D. The discrepancy between measurement and simulation is attributed to the finite aperture of the beam, which perturbs its outer rings (i.e., its OAM reservoir). Similar to the case of monotonic OAM growth and decay, here, the initial and final beam sizes (at *z* = 1 cm and *z* = 40 cm) are not the same despite carrying equal charge of ℓ = 2. To reconcile this behavior, recall that, in order for a beam with linearly evolving OAM to modify its charge ℓ, the phasefront should be continuously deformed with propagation. The phase dependence of the beam with propagation-dependent OAM is expressed as

$$e^{{\mathrm{iK}}_{z}z}∼e^{i\left(k_{z,0}\right)z+i\ell_{\text{eff}}\left(z\right)\phi }$$ (5)

where *K*_*z*_ is the effective longitudinal wave vector of the envelope, which now has two contributing terms: (i) a propagation phase term, *e*^*ik*_*z*,0_*z*^, as expected from a normal Bessel beam with fixed OAM, and (ii) an additional Gouy phase factor $e^{i\ell_{\text{eff}}\left(z\right)\phi }$, which stems from the OAM evolution. Hence, points along the azimuthal direction (ϕ) of the wavefront acquire a *z*-dependent phase Φ(*z*) = ℓ_eff_(*z*)ϕ. The slope of this phase with respect to *z* is reminiscent of an effective wave vector or momentum, which we denote as *k*_B_. Therefore, although a vortex beam with constant OAM accumulates phase following ∼*e*^*ik*_*z*,0_*z*^, a vortex beam with linearly evolving OAM experiences a *k*-shift of *k*_B_ = ∂Φ(*z*)/∂*z* such that its effective longitudinal wave vector becomes *K*_*z*_ = *k*_*z*,0_ + *k*_B_. Because the temporal frequency of the beam is unchanged and $K_{z}^{2}+k_{\rho }^{2}={\left(\omega /c\right)}^{2}$, then a shift in *K*_*z*_ mandates a subsequent shift in *k*_ρ_ from momentum conservation. This wave vector perturbation translates to a change in the transverse size of the beam. Therefore, the beam’s size is perturbed depending on the sign and magnitude of ℓ_eff_(*z*). This is consistent with the measured profiles of Fig. 3 (A and C), encircled with dashed lines. To better visualize this effect, 1D cuts of the transverse profile are plotted in Fig. 3 (E to G). These plots suggest that the linear growth and decay of OAM is associated with red and blue shifts in the spatial frequency of the beam, respectively. This dependence stems from our phase convention (*e*^*ik*_*z*,0_*z*^) and would be reversed if the *e*^−*jk*_*z*,0_*z*^ convention is adopted instead. A vortex beam with constant charge does not experience such a perturbation in its *k*-vector (see fig. S4). An analog of this additional phase factor (and associated *k*-shift) has been observed in the case of vector beams with *z*-dependent polarization evolution (*60*). The latter has been reconciled as a Berry phase factor, which is acquired as the beam adiabatically modifies its spin angular momentum with propagation. Similarly, here, as the vortex beam undergoes an adiabatic evolution in its parameter space (ℓ), to deform its topology, it acquires the additional Gouy phase factor: $e^{i\ell_{\text{eff}}\left(z\right)\phi }$ (*61*), which, in turn, is a special class of Berry phase (*62*–*64*). The latter is accumulated whenever a quantal system in an eigenstate slowly evolves in its parameter space as detailed in ref. (*63*). In the following, we expand the scope of our method and demonstrate vortex beams with quadratic evolution of OAM.

### Quadratic evolution of OAM

The slope and curvature of the OAM evolution can be designed on demand using our approach. By allowing the topological charge to experience a quadratic chirp along the optical path, we create a vortex beam with optical rotatum. To demonstrate this, we consider a vortex beam with a helical phasefront that follows $∼e^{i100z^{2}\phi }$ dependence. This target phase profile is chosen by design. It allows the vortex beam to reach ℓ = 1, 2, 3, and 4 at *z* = 10, 14, 17, and 20 cm, respectively. Setting this as the target function in Eq. 1 with *L* = 50 cm yields the following closed form expression for ${\tilde{A}}_{n}\left(\phi \right)$

$${\tilde{A}}_{n}\left(\phi \right)=i\frac{\sqrt{\pi }e^{-\frac{{\text{in}}^{2}\pi^{2}}{25\phi }}\text{erf}\left(\frac{2\pi n}{\sqrt{100i\phi }}\right)}{\sqrt{100i\phi }}+i\frac{\sqrt{\pi }\text{erf}\left(\frac{40\phi -2\pi n}{\sqrt{100i\phi }}\right)e^{-\frac{{\text{in}}^{2}\pi^{2}}{25\phi }}}{\sqrt{100i\phi }}$$ (6)

These are the complex amplitude terms of the Bessel beams in Eq. 3. Solving the latter (Eq. 3) at *z* = 0 provides the target profile to be generated by the SLM.

**Fig. 4.**
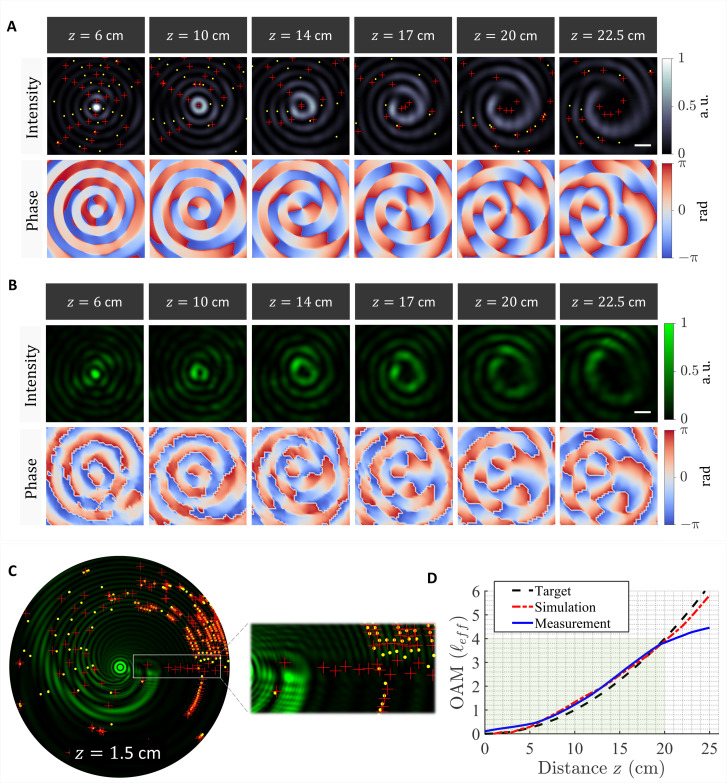
Optical vortex beams with quadratic evolution of topological charge: Optical rotatum. (**A**) Simulated transverse profiles (intensity and phase) of a vortex beam whose OAM locally grows in a quadratic manner along the optical path. The beam acquires a helical phase, increasing its OAM continuously from ℓ = 0 to ℓ = 5 over a range of 22.5 cm. The red and yellow markers denote phase singularities of opposite handedness (positive and negative helicity, respectively). Scale bar, 100 μm. (**B**) Measured 2D intensity and phase profiles at the *z* planes in (A). (**C**) Intensity profile of the vortex beam at *z* = 1.5 cm. The inset depicts a close up exhibiting a line of phase singularities feeding the beam’s center. (**D**) Comparison between the target, simulated, and measured local charge (ℓ_eff_) showing its quadratic dependence on *z*.

Figure 4A shows the simulated 2D intensity and phase profiles of the resulting beam at different propagation distances. In this case, the wavefront evolves from an approximately flat (ℓ = 0) to a helical (ℓ = 5) profile, in a continuous manner, as the beam propagates. The red and yellow markers denote the positive and negative phase singularities. The precise movement of these singularities underpins the evolution of the topological charge at different *z* planes. For instance, at each *z* plane, an additional singularity is accumulated inside the central ring of the beam. The singularities approach the beam’s center via the dark fringes in its vicinity. The measured intensity and phase profiles are shown in Fig. 4B and movie S4 and are in very good agreement with the calculated ones. The initial beam profile (at *z* = 1 cm) is depicted in Fig. 4C. Similar to the case of vortex beam with linear evolution of OAM, here, we observe a few key features: (i) a region of bifurcation at the interface between the fast and slow spatial oscillations of the Bessel beam. This dislocation arises due to the local mismatch in the spatial frequency along the azimuthal direction (see, for example, Fig. 1C). (ii) A chain of phase discontinuities (i.e., off-center isolated singularities) of alternating signs, which approach the beam’s center via a dark intensity line. This is a well-known signature of beams with fractional topological charge as previously discussed.

In addition, a distribution of phase singularities is observed at the outer peripherals of the beam. Its inner contour follows a growing logarithmic spiral and is reminiscent of the shape of a Nautilus seashell as illustrated in fig. S9 (*65*). This spiral signature arises due to the azimuthal chirp in the spatial frequency of the beam (Fig. 1C), which introduces zero crossings (i.e., phase singularities) along the azimuthal direction. These phase singularities or discontinuities trace a spiral path in space whose curvature depends on the azimuthal chirp rate in spatial frequency. The spiral curvature also depends on the encoded OAM evolution, i.e., whether it is linear or quadratic. This is because the outer rings of the Bessel beam take the role of the momentum reservoir, which stores local singularities (isolated vortices) that gradually move into the center of the beam to change its topological charge. The growth factor of the spiral in case of rotatum is close to Fibonacci’s golden ratio, which is also close to our target quadratic growth for ℓ_eff_ where $e^{i\ell_{\text{eff}}\phi }∼e^{i\phi 100z^{2}}$. As the beam propagates, this spiral signature becomes less pronounced as the isolated singularities disperse across the transverse profile of the beam.

To confirm the quadratic evolution of OAM, we calculated and measured the local effective topological charge (ℓ_eff_) of the beam as a function of propagation distance. A comparison between the target and realized profiles is depicted in Fig. 4D. From this result, it can be inferred that the OAM is locally chirped along the optical path following the desired parabolic profile. Notably, this behavior occurs only locally at the center of the beam. The global OAM is always conserved owing to the engineered movement of the phase singularities across the beam (see fig. S6) (*37*, *43*). We observe a discrepancy between the measured and simulated evolution of ℓ_eff_ beyond a propagation distance of 20 cm. This deviation is due to the finite aperture size of our beam, which allows it to be nondiffracting for a limited distance. Beyond this distance, the beam diffracts and disperses its OAM content outside our observation window. Note that, in analogy with the linearly evolving OAM, the quadratic evolution here is also associated with an accumulated Gouy phase factor ($e^{i\phi 100z^{2}}$), which perturbs the spatial frequency of the beam. In this case, the *k*_*z*_-vector of the ensemble experiences a linear chirp along the optical path. This effect is further examined in fig. S5. Furthermore, we simulated another scenario of optical rotatum with a larger number of Bessel beams, which better approximate the parabolic evolution of OAM (fig. S6). Last, the temporal analog of optical rotatum can be realized using Bessel beams of different wavelengths such that their beating gives rise to a time varying OAM. This will be the subject of future work.

## DISCUSSION

We demonstrated a property of light, optical rotatum, in which an optical vortex experiences a chirp in its OAM as it propagates. We showed that such an adiabatic deformation of the topological charge is associated with a Gouy phase factor that perturbs the spatial frequency of the beam. Our approach is fully analytical and can be applied to other regions of the electromagnetic spectrum in addition to ultrasound and electron beams. We stress that, in general, the OAM content of a beam is not necessarily proportional to the strengths of the vortices it contains (*66*, *67*). However, the use of the topological charge as a direct measure of OAM was valid in our analysis because our ℓ_eff_ was calculated over a small observation window at the center of the beam in which the singularities have the same polarity/sign and therefore do not cancel out. On the basis of this analysis, vortex beams with linear and quadratic chirp in their OAM were demonstrated. Structured waves of this kind may inspire new directions in science and technology. It advances the field of singular optics by enabling topologically complex states of light, which, in turn, can lead to many interesting phenomena in quantum and classical optics (*68*, *69*). More specifically, spatially chirped vortex beams can be used in depth sensing, metrology, laser plasma physics, and free space communications. Furthermore, as an unexplored property of light, rotatum may reveal a rich class of optical forces, which can be exploited in light-matter interaction, micromanipulation, valleytronics, and spintronics both in the near- and far-field regimes.

It is noteworthy that, given our choice of Bessel functions as the OAM modes, our vortex beams are characterized by a nondiffracting and self-healing behavior (*54*), which is desirable in many applications. Moreover, the dimensions of our beams and their propagation range can be readily modified by changing the aperture size of the devices and the cone angles of the OAM modes following the same design considerations of axicons (*54*). Although we primarily focused on scalar vortex beams, our approach can be extended to vector beams with spatially varying polarization states, enabling rich spin-orbit interactions in free space, which can inspire multidimensional information encoding and multiplexing (*70*–*73*). This will be the subject of future work. Last, the multidisciplinary nature of angular momentum and singularity engineering across different fields may inspire related research efforts in the areas of microfluidics, acoustics, and pattern formation, to name a few. Therefore, we thus envision this work to enrich the science and applications of wave physics, singular optics, structured light, and beyond.

## MATERIALS AND METHODS

### Engineering the vortex beams

The spatially evolving optical vortex beams discussed in this work were constructed from the superposition of copropagating Bessel vortex beams given by

$$\psi \left(\rho ,\phi ,z,t\right)=\sum_{n=-N}^{n=N}{\tilde{A}}_{n}\left(\phi \right)J_{0}\left(k_{\rho ,n}\rho \right)e^{{\mathrm{ik}}_{z,n^{z}}}e^{-i\omega t}$$ (7)

where *k*_ρ,*n*_ and *k*_*z*,*n*_ denote the transverse and longitudinal components of the wave vectors, respectively. For the case of optical vortex beams with linear evolution of OAM, we set *N* = 8, which yields 17 Bessel beams in the superposition. The 17 Bessel beams are equally spaced in *k*_*z*_-space by a separation of 2π/*L*. The central *k*_*z*,0_ was set to 0.999991 *k*_0_, and the resulting beam extended for a range *L* = 50 cm. These parameters dictate the degree of paraxiality of the generated beam. For the case of vortex beams with quadratic evolution of OAM, we reduced *k*_*z*,0_ to 0.99998 *k*_0_ and, in turn, increased *N* to 18. This allowed us to include 37 Bessel beams in the superposition, providing a more accurate reconstruction of the target evolution. To ensure effective generation of the target vortex beam profile over the propagation range *L*, with minimal diffraction, the aperture radius of the wavefront shaping device should satisfy the following criterion (*37*)

$$R_{\text{aperture}}=L\sqrt{{\left[\frac{k_{0}}{{\left(k_{z,n}\right)}_{\text{max}}}\right]}^{2}-1}$$ (8)

### Evaluating the effective topological charge

The topological charge, ℓ, is typically a quantized value. Nevertheless, given a local field distribution, one can calculate an effective topological charge (ℓ_eff_) by integrating the OAM density and energy densities of that field over a localized region (i.e., a subregion of the beam). The resulting quantity (ℓ_eff_) can be integer or fractional. To derive this calculation, we follow the procedure adopted in refs. (*58*, *59*). We start by defining a vector potential **A** in the cartesian coordinates under the Lorentz gauge to represent our linearly polarized field ψ from Eq. 3

$$A=\psi =u\left(x,y,z\right)e^{i\left(k_{z}z-\omega t\right)}\hat{x}$$ (9)

The electric and magnetic field components of our complex scalar field are given by

B=∇×A (10)

$$E=\frac{{\mathrm{ic}}^{2}}{\omega }\nabla \times B$$ (11)

This yields the following expressions for the electric and magnetic fields (*58*, *59*)

$$B={\mathrm{ik}}_{z}e^{i\left(k_{z}z-\omega t\right)}\left(u\hat{y}+\frac{i}{k_{z}}\frac{\partial u}{\partial y}\hat{z}\right)$$ (12)

$$E=i\omega e^{i\left(k_{z}z-\omega t\right)}\left(u\hat{x}+\frac{i}{k_{z}}\frac{\partial u}{\partial x}\hat{z}\right)$$ (13)

Here, we implemented the paraxial approximation and neglected the derivatives of the *z* components of the fields. Next, we calculate the time average of the Poynting vector from

$$P=c^{2}\epsilon_{0}\langle E_{\text{real}}\times B_{\text{real}}\rangle$$ (14)

The real parts of the fields are readily expressed as follows

$$E_{\text{real}}=\frac{1}{2}\left(E+E^{*}\right)$$ (15)

$$B_{\text{real}}=\frac{1}{2}\left(B+B^{*}\right)$$ (16)

By substituting Eqs. 15 and 16 and Eqs. 12 and 13 in Eq. 14 and canceling the time-harmonic terms, the time-averaged Poynting vector becomes

$$\begin{array}{c} P=\frac{c^{2}\epsilon_{0}}{4}(E\times B^{*}+E^{*}\times B)=\\ \frac{\epsilon_{0}\omega }{4}\left\{i\left(u\nabla u^{*}+u^{*}\nabla u\right)+2k_{z}{|u|}^{2}\hat{z}\right\} \end{array}$$ (17)

Here, ϵ_0_ is the free space permittivity (8.854 × 10^−12^ F/m), ω is the angular frequency, and *c* is the speed of light in vacuum. For a field with dependence $u\left(\rho ,\phi ,z\right)=u_{0}\left(\rho ,z\right)e^{i\ell \phi }$, in the cylindrical coordinates, it can be shown that the ϕ component of the linear momentum density $\epsilon_{0}{\langle E\times B\rangle }_{\phi }=\epsilon_{0}\omega \ell {|u|}^{2}/\rho$. Its cross product with **ρ** gives the OAM density. Hence, the OAM density along the beam’s axis, j_**z**_, is evaluated from the cross product of the radius vector and the azimuthal component of the Poynting vector

$$j_{z}=\frac{1}{c^{2}}\left(\rho \times P_{\phi }\right)$$ (18)

where **ρ** is the radius vector. In addition, the energy density of the beam is given by the speed of light multiplied by the linear momentum density: $w=c\epsilon_{0}{\langle E\times B\rangle }_{z}=c\epsilon_{0}\omega k{|u|}^{2}$. Hence, the ratio between the OAM density and energy density is given by

$$\frac{j_{z}}{w}=\frac{\ell }{\omega }$$ (19)

Integrating j_*z*_ over a given transverse cross section of the beam yields the OAM associated with that area, denoted as L_*z*_ such that

$$L_{z}=\int \int j_{z}\text{ρ}d\text{ρ}d\phi$$ (20)

where it is understood that L_*z*_ is evaluated per unit length. To evaluate the effective charge over a given subregion of the beam, we need to normalize L_*z*_ by the total energy of the beam over the same region. The total energy per unit length, W, is obtained by integrating w over the transverse cross section of the beam such that

$$W=c\epsilon_{0}\int \int {\langle E\times B\rangle }_{z}\text{ρ}d\text{ρ}d\phi$$ (21)

Normalizing L_*z*_ by W yields the quantity (ℏℓ)/(ℏω), which is proportional to the mean OAM per photon, scaled by 1/(ℏω); see, for example, Eq. 19 above and Eqs. 2.8 and 2.18 in Allen *et al.* (*9*). Therefore, in the paraxial regime, the effective topological charge (ℓ_eff_) can be calculated from the ratio

$$\frac{L_{z}}{W}=\frac{\ell_{\text{eff}}}{\omega }$$ (22)

Here, ℓ_eff_ is interpreted as a measure of the OAM per photon and can acquire a noninteger value. By calculating this quantity over a small window around the beam center (excluding its outer rings), we obtain the local ℓ_eff_, which can vary with propagation because new singularities can enter/exit that local window at each *z* plane. However, ℓ_eff_ remains conserved at each *z* plane if the integration is performed across the entire cross section of the beam including all the outer rings. In our calculations, the limits of integration of the circular aperture were set to a diameter of 1.9 mm for evaluating the global OAM. The dependence of this calculation on the choice of the limits of integration is detailed in fig. S2.

### Experimental setup

To generate the desired vortex beams, we used a reflective phase-only SLM (Santec SLM-200) with a resolution of 1920 by 1200 pixel and 8-μm pixel pitch. We started by converting the 2D complex amplitude profile of Eq. 3 to a phase-only computer-generated hologram, to be compatible with our SLM, following the method outlined in ref. (*74*). Our measurements were obtained using a 532-nm laser source (Novanta Photonics, Ventus Solid State CW laser) with the standard 4-*f* holography setup depicted in Fig. 2A. The laser beam was first expanded and collimated (using a 40X objective lens, a 100-μm pinhole, and a 50-cm lens) onto the reflective SLM screen. The desired complex amplitude spectrum was generated at the Fourier plane (*k*-space) of the SLM using the first lens in Fig. 2A. The spectrum was then filtered from the zeroth and higher diffraction orders with an iris. Spatial filtering in *k*-space with an iris is a requirement for allowing a phase-only SLM to realize complex-amplitude modulation on the desired diffraction order while relying on the higher diffraction orders to act as loss channels. After filtering, we used a second lens to perform an inverse Fourier on the filtered spectrum, transforming it into the desired vortex beam at the focal plane (*z* = 0) to be recorded with a charge-coupled device (CCD) camera (Thorlabs DCU224C, 1280 by 1024 resolution). The CCD was mounted on a translation stage (Thorlabs LTS150) to capture the beam’s evolution with steps of 0.25 mm along its optical path. From these *z*-dependent intensity measurements, the phase profile was also retrieved following the SBMIR technique (*55*). To limit the noise effects during data acquisition, we used an adapted version of the flat fielding procedure described in the European Machine Vision Association’s Standard 1288 (*75*). The following adapted flat fielding procedure was previously performed in other work (*76*). Profiles of the sensor’s dark image and pixel-wise responsivity were captured and applied to each measurement taken. The same 532-nm laser source was used as an input for sensor characterization. The power of the laser was driven from 0 to 100% in 5% steps, where 100% is the illumination level required to saturate the sensor at the exposure times used to capture each dataset. The 0% illumination image was taken as the dark current response of the sensor. To limit shot noise effects, between 50 and 70 frames were averaged per frame. Each pixel on the sensor then had its responsivity curve fit to the irradiance witnessed by the independent reference photodetector. The responsivity curve was inversely applied to the images before effective topological charge was calculated.

## Supplementary Materials

### The PDF file includes:

Sections S1 to S9 Figs. S1 to S9 Legends for movies S1 to S4
