# Supplementary material for: Rotatum of light
Source: Sci Adv. 2025 Apr 11;11(15):eadr9092. doi: 10.1126/sciadv.adr9092 (PMC11988407; doi:10.1126/sciadv.adr9092)
Supplement: Supplementary file 1 — Sections S1 to S9 Figs. S1 to S9 Legends for movies S1 to S4 [file sciadv.adr9092_sm.pdf]

Science Advances
AAAS

Supplementary Materials for

Rotatum of light

Ahmed H. Dorrah *et al.*

Corresponding author: Ahmed H. Dorrah, dorrah@seas.harvard.edu; Federico Capasso, capasso@seas.harvard.edu

Sci. Adv. **11**, eadr9092 (2025)
DOI: 10.1126/sciadv.adr9092

The PDF file includes:

Sections S1 to S9
Figs. S1 to S9
Legends for movies S1 to S4

Other Supplementary Material for this manuscript includes the following:

Movies S1 to S4

1 Error Analysis

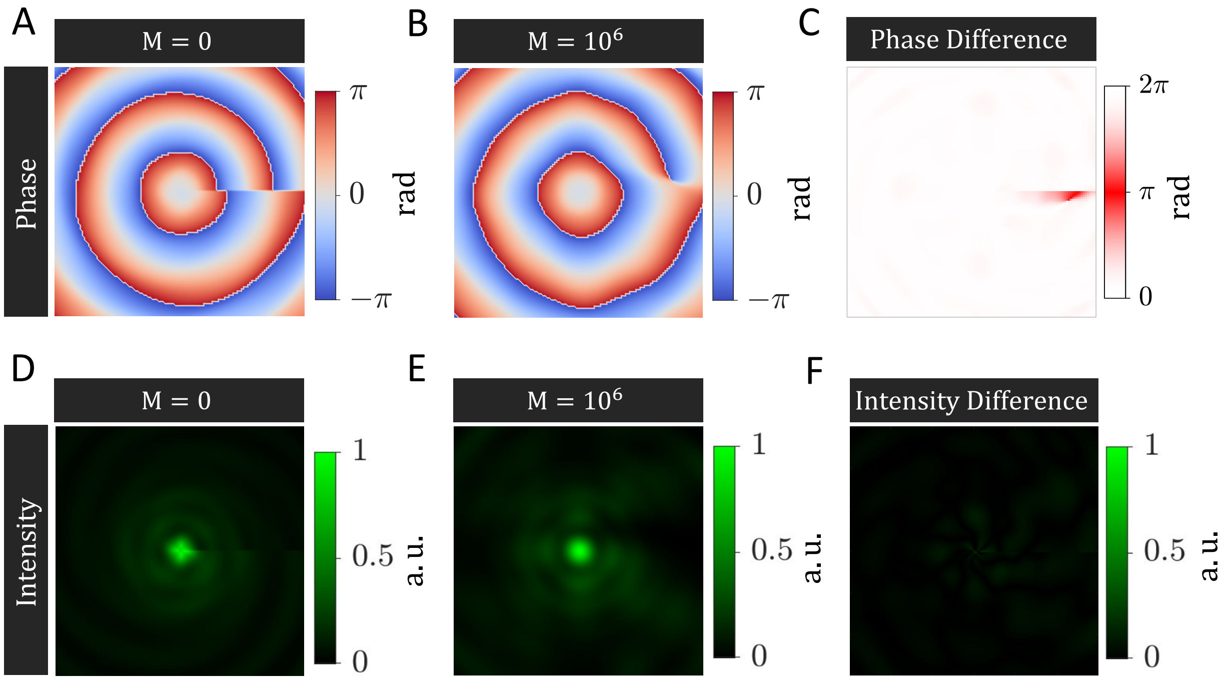

Figure S1: Comparison between the phase and intensity profiles of the vortex beam with linearly evolving OAM using the exact and approximate solution to the wave equation. **(A)** Phase profile at $z = 0$ under the approximation $\tilde{A}_{n,m} = \tilde{A}_n$ and $M = 0$. **(B)** Phase profile at $z = 0$ under the exact solution to the wave equation, Eq. (3) of the main manuscript, with $M = 10^6$ ($2M + 1$ Fourier terms). **(C)** Phase error calculated as the point-by-point absolute difference between the phase profiles in (A-B). **(D)** Intensity profile at $z = 0$ for the beam under the approximation $\tilde{A}_{n,m} = \tilde{A}_n$ and $M = 0$. **(E)** Intensity profile at $z = 0$ under the exact solution to the wave equation with $M = 10^6$ ($2M + 1$ Fourier terms). **(F)** Intensity error calculated as the point-by-point absolute difference between the intensity profiles in (A-B). The mean error in intensity and phase over the shown window (500 μm) is in the order of 1%. The discrepancy is primarily observed at the phase dislocation (sharp horizontal edge at the right side of the beam) and is less pronounced at longer propagation distances. This mismatch can be mitigated by including a larger number of Fourier terms in Eq. (3).

2 OAM Calculation

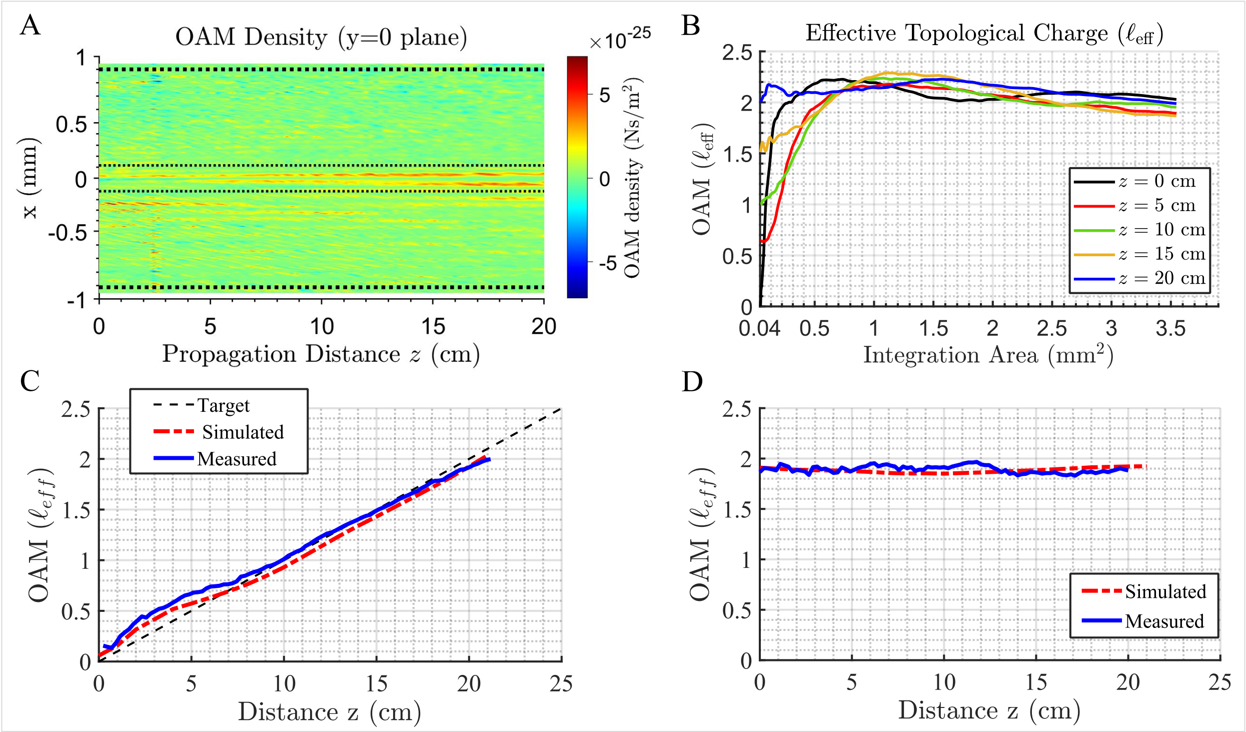

Figure S2: Evaluating the effective topological charge ℓ_{eff} and OAM of a beam with linearly evolving charge as a function of propagation distance. **(A)** Longitudinal profile of OAM density calculated based on Eq. (18) of the main text, applied on the measured beam profile. The dotted black lines denote the limits of integration for calculating the local (200 μm) and global (1.8 mm) OAM. **(B)** Effective topological charge (ℓ_{eff}) of the beam in (A) evaluated based on Eq. (22) of the main text. The calculation is performed at 5 different z-planes. For each z-plane, the integration limit (area) is varied to capture both the local OAM around the beam's center, considering 0.04 mm^2 integration area, and the global OAM, considering the outer rings of the beam. **(C)** Local OAM evolution considering a small integration window with 200 μm side length around the center of the beam. **(D)** Global OAM considering the entire cross section of the beam; i.e., integration window with 1.8 mm side length. These results suggest that the effective charge of the beam varies locally while keeping the global OAM conserved at each z-plane.

3 Linear Growth and Decay of OAM

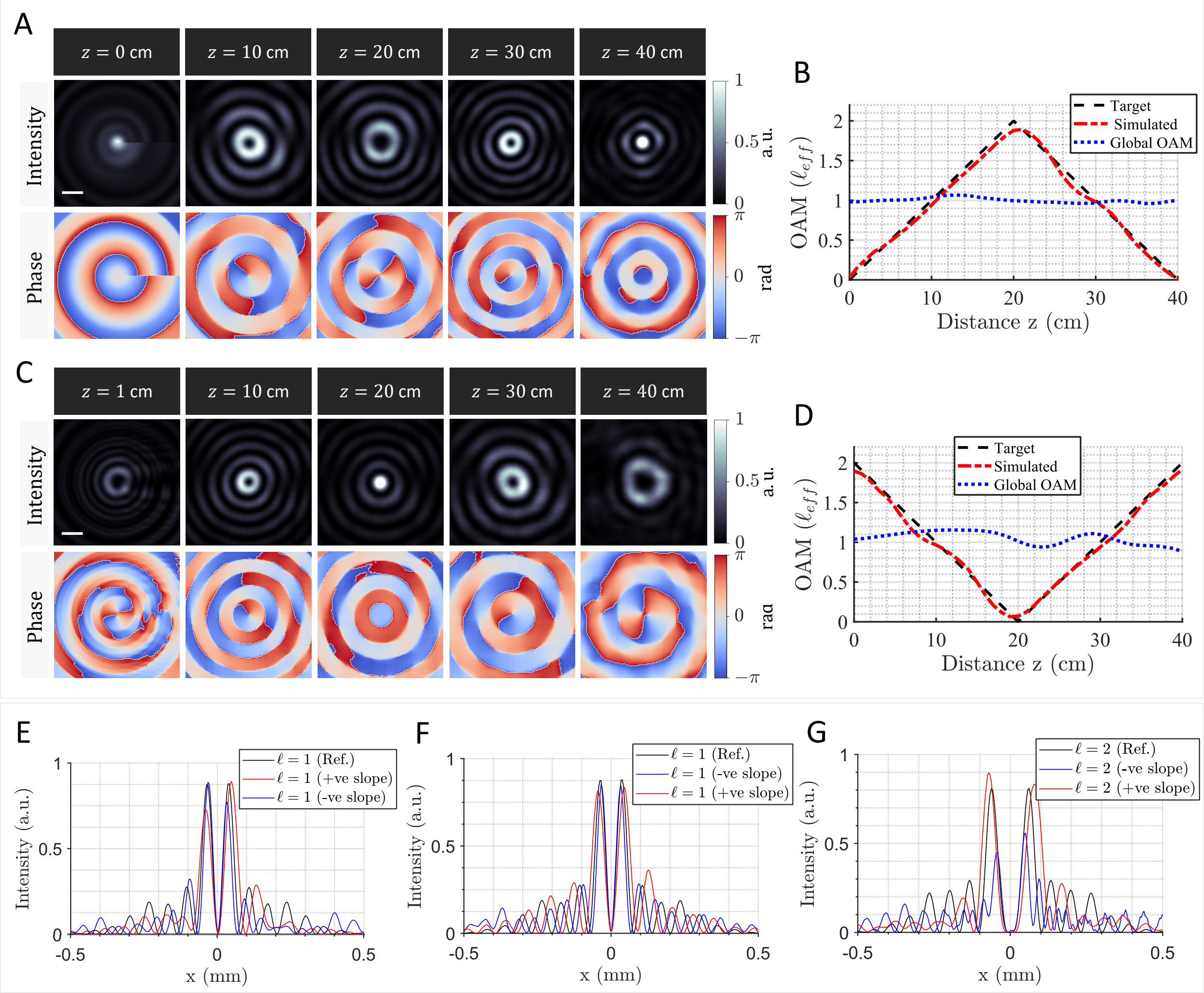

Figure S3: Non-monotonic spatial self-torque. **(A)** Simulated 2D transverse profiles of a vortex beam which changes the strength of its vorticity as it propagates. Here, the topological charge (ℓ) continuously increases from $\ell = 0$ to $\ell = 2$ before it decays to $\ell = 0$. Scale bar is 100 μm. **(B)** Simulated evolution of effective topological charge (ℓ_{eff}) as a function of propagation distance. **(C)** Simulated 2D transverse profiles of a vortex beam in which ℓ initially decays from $\ell = 2$ to $\ell = 0$ in a linear manner before growing to $\ell = 2$. **(D)** The corresponding evolution of ℓ_{eff}. This adiabatic change in topology is associated with a perturbation in the propagation constant (the spatial frequency) of the beam. **(E)** Cross sectional 1D cuts of the vortex beam of (A) at $z = 10$ cm and $z = 30$ cm compared to a vortex beam of constant charge $\ell = 1$. Although ℓ is the same in the three profiles, the beams dimensions are slightly perturbed depending on the growth (+ve slope) or decay of their charge ℓ. **(F)** Comparison between the 1D cuts of the beam in (C) at $z = 10$ cm and $z = 30$ cm as well as a vortex with constant charge $\ell = 1$. The perturbation in size is reversed compared to (E). **G)** Comparison between the 1D cuts of the beam in (C) at $z = 1$ cm and $z = 40$ cm as and a vortex with constant charge $\ell = 2$. The shift in the spatial frequencies occurs due to the modulation of ℓ, alluding to an underlying Gouy phase $\sim e^{i\ell(z)\phi}$.

4 Gouy Phase in Linear OAM Evolution

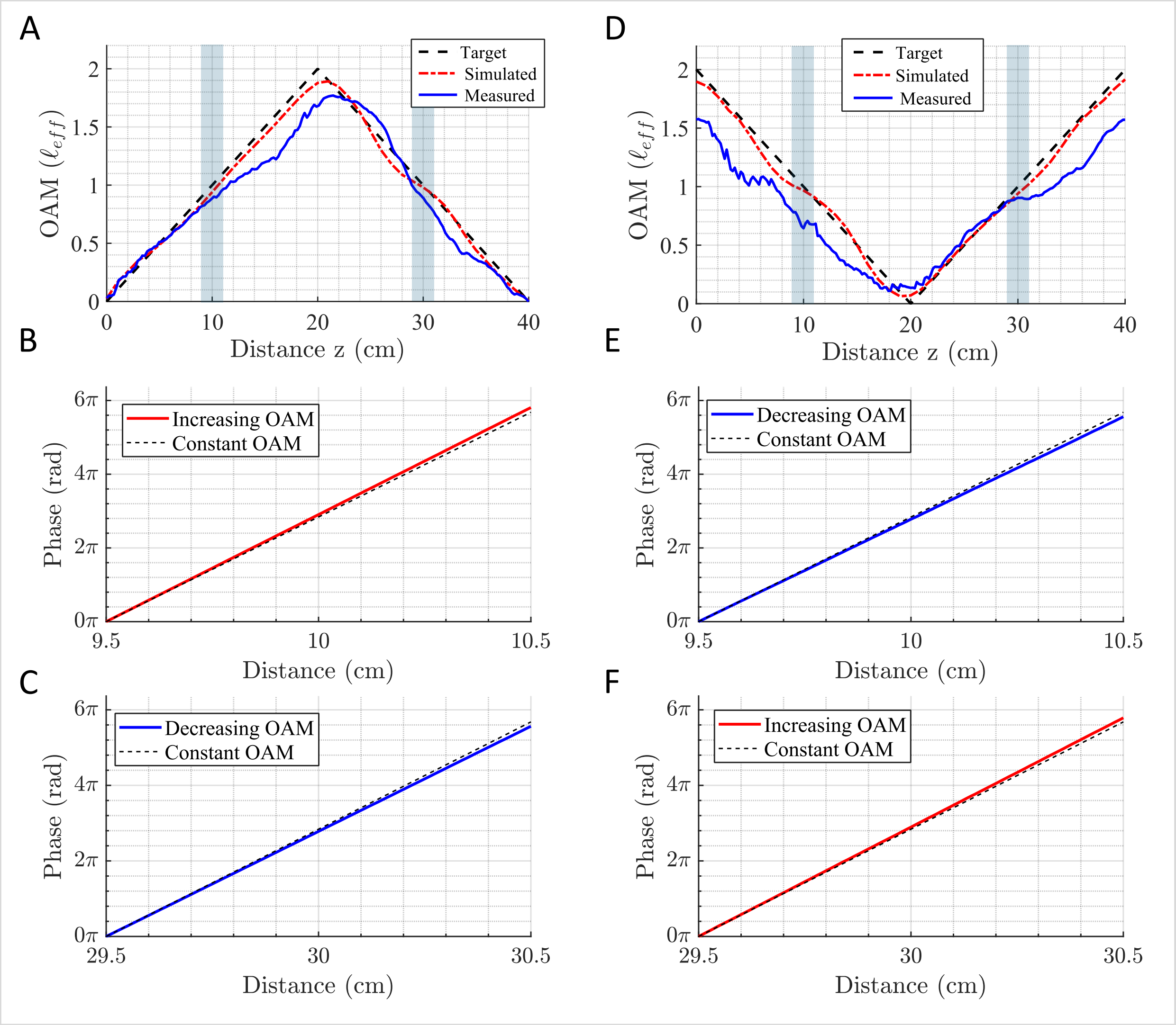

Figure S4: Phase accumulation in vortex beams with linearly evolving OAM in comparison to vortex beams with constant OAM. (A) Evolution of OAM in a vortex beam whose topological charge grows then decays linearly as a function of distance. **(B)** Accumulated phase of the beam in (A) over a 1-cm distance around 10 cm in comparison to a vortex beam of the same charge with constant OAM. **(C)** Accumulated phase of the beam in (A) over a short distance around 30 cm in comparison to a vortex beam of the same charge with constant OAM. **(D)** Evolution of OAM in a vortex beam whose topological charge decays then grows linearly along the optical path. **(E)** Accumulated phase of the beam in (D) over a short distance around 10 cm in comparison to a vortex beam of the same charge with constant OAM. **(F)** Accumulated phase of the beam in (D) over a short distance around 30 cm in comparison to a vortex beam of the same charge with constant OAM. The acquired phase is a class of Gouy phase which amounts to a shift in the longitudinal wavevector (k_z) of the beam.

5 Gouy Phase in Quadratic OAM Evolution

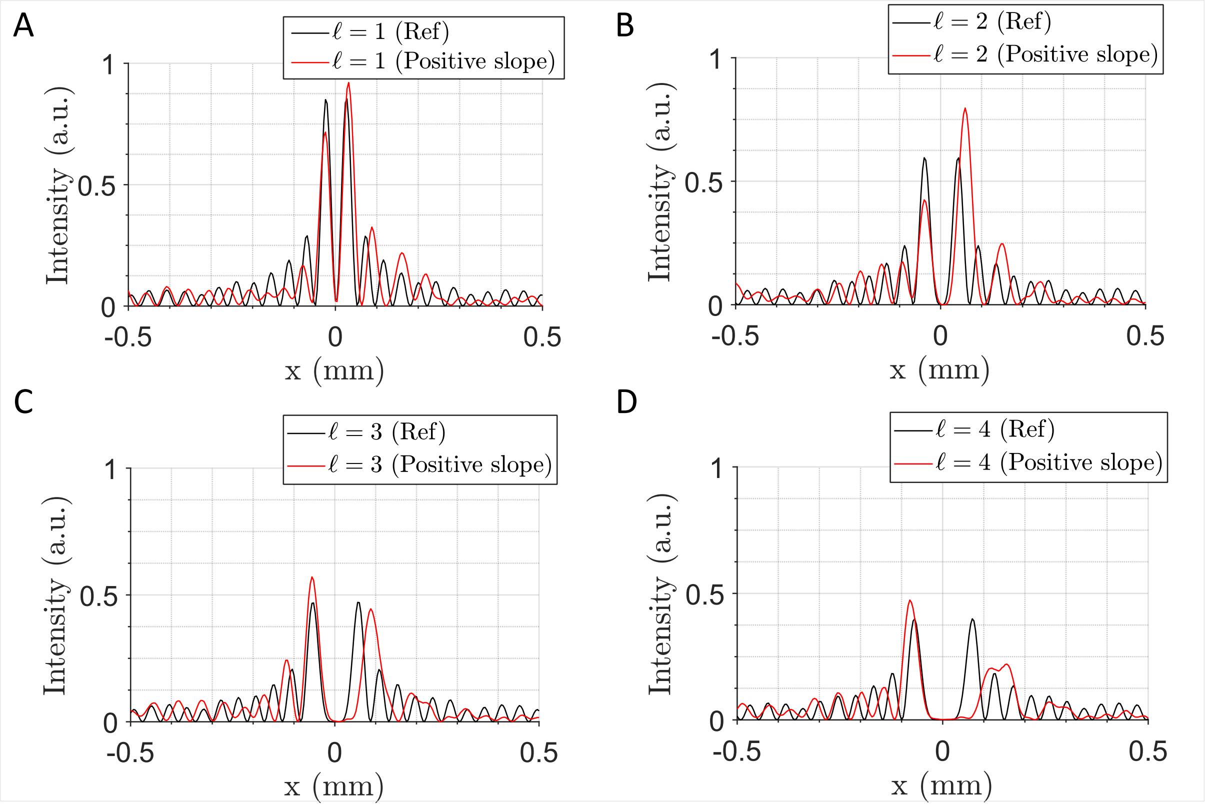

Figure S5: Rotatum and spatial frequency shifts. Simulated 1D transverse cuts of the intensity profile for the vortex beam of Fig. 4 in comparison to a vortex beam with constant OAM, ℓ. Here, the spatially varying vortex changes its OAM in a quadratic manner along the optical path. Its profile is compared with a reference vortex of fixed ℓ at four different z-planes: $z = 10$ cm (**a**), 14 cm (**b**), 17 cm (**c**), and 20 cm (**d**). It is observed that the spatially evolving vortex experiences a red shift in its transverse spatial frequency (k_ρ) which can be inferred from the slight perturbation (stretching) in the lateral dimensions of the beam compared to a reference vortex. The underlying mechanism of this k-shift is an accumulated propagation-dependent Gouy phase $\sim e^{i\ell(z)\phi}$.

6 Quadratic Growth of OAM

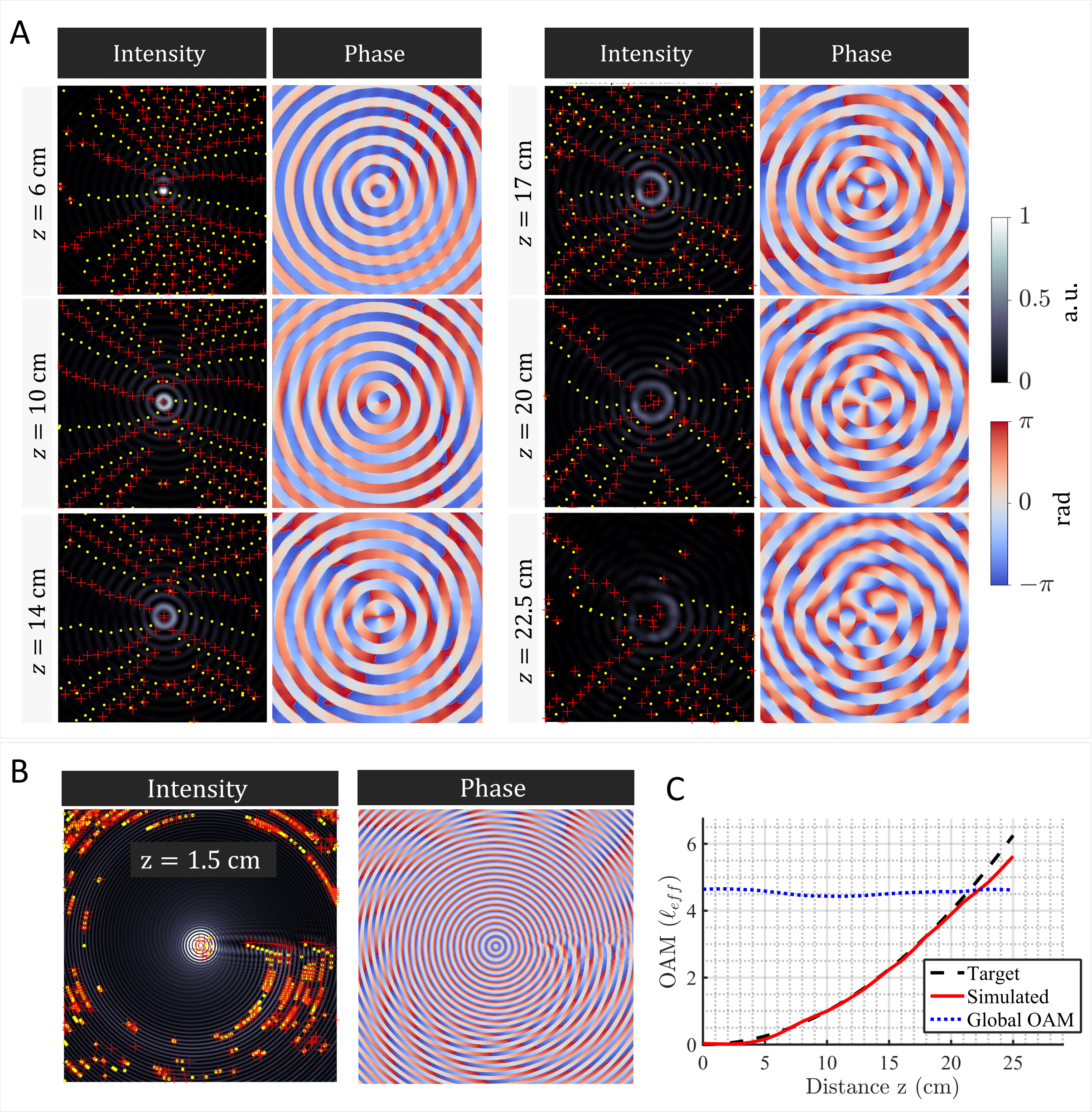

Figure S6: Optical vortices with quadratic evolution of topological charge: optical rotatum. (A). Simulated transverse profiles (intensity and phase) of a vortex beam whose OAM locally grows in a quadratic manner along the optical path. Here, $N = 45$ which yields 91 co-propagating Bessel vortices with longitudinal wavevectors centered at $k_{z,0} = 0.99995K_0$. The resulting vortex beam acquires a helical phase, increasing its OAM continuously from $\ell = 0$ to $\ell = 5$ over a range of 22.5 cm. The markers denote the positive and negative phase singularities. **(B)** The 2D intensity and phase profile of the vortex at $z = 1.5$ cm. A line of phase singularities feeds the beam's center. Its contour is reminiscent of a seashell structure or a logarithmic spiral. **(C)** Target and simulated local charge ($\ell_{\rm eff}$) showing its quadratic dependence on z.

7 Longitudinal Profiles

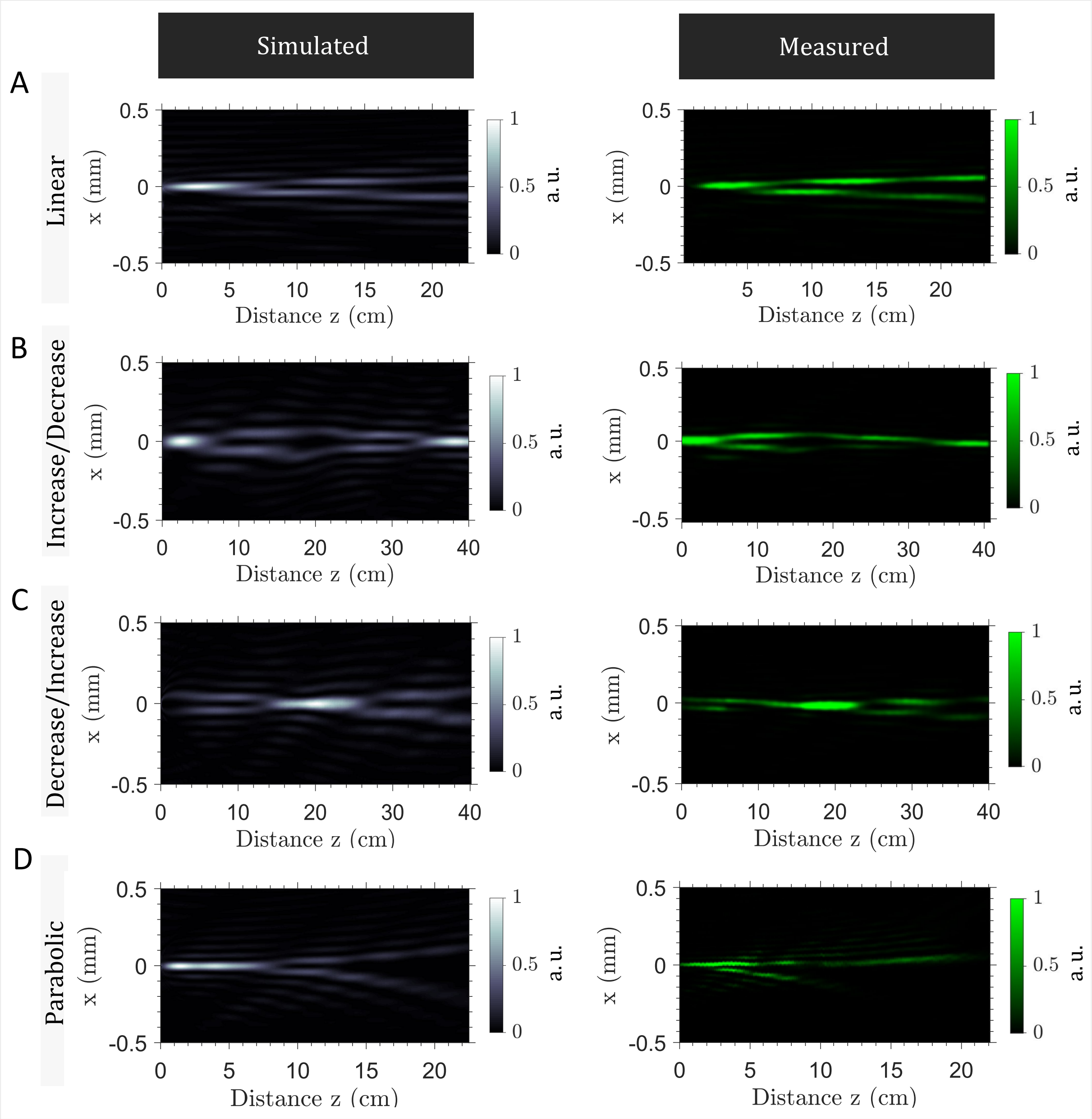

Figure S7: Simulated and measured longitudinal intensity profiles of four different vortex beams. (A) Linearly growing OAM. **(B)** Linearly growing then decaying OAM. **(C)** Linearly decaying then growing OAM. **(D)** Quadratically growing OAM.

8 Phase Retrieval Algorithm

The 2D phase profiles of the generated vortices were obtained at each z position using a modified version of the single-beam multiple-intensity reconstruction (SBMIR) method (52). This is an indirect measurement since the CCD camera used in our experiment can only detect intensity information. Note that phase information can be measured using a wavefront sensor, for e.g., a Shack Hartman microlens configuration, on the expense of low resolution. Instead the SBMIR method allows us to accurately retrieve the phase information by recording at least two intensity patterns of the object at different z positions. By applying an iterative calculation on these intensity measurements, the 2D phase profile can be reconstructed. The workflow is as follows: first, $n + 1$ intensity patterns are recorded using a CCD camera at equidistant positions separated by Δz. Next, a constant phase ($\phi_0 = 0$) is assumed for the intensity pattern at the origin ($z_0 = 0$). This frame acts as a reference. Hence, it is possible to express the wavefront in the form $\sqrt{I_0}e^{i\phi_0}$. This field is then propagated from $z_0 = 0$ to $z_1 = z_0 + \Delta z$ using the Rayleigh–Sommerfeld equation.

The complex amplitude at z_1 given by $A_1e^{i\phi_1}$ is rearranged as follows: the phase term $e^{i\phi_1}$ is combined with the square root of the measured intensity so that the new complex wave front at the position $z1 = z0 + \Delta z$ is given by $\sqrt{I_1}e^{i\phi_1}$ ready to be propagated to the new position $z_2 = z_0 + 2\Delta z$. The same procedure is so repeated for all the other intensity patterns until the last one at the position $z_n = z_0 + n\Delta z$. Once this last complex amplitude has been obtained, the same process is repeated using back-propagation Rayleigh–Sommerfeld relation.

The aforementioned method is depicted in Fig. S8. We assigned the measured intensity profile at $z = 0$ cm as the boundary condition for the forward propagation. The initial 2D phase profiles for all the planes were set to random values between 0 and 2 π. These profiles were then updated by the phase of the forward or backward propagated fields. At each plane, the propagated RMS intensity was then replaced by the measured RMS intensity at that plane. The complex amplitude (including the retrieved phase) was propagated one additional step, again replacing the intensity part with the measurement while updating the phase part. Each cycle was repeated 2000 times or until convergence was reached. Convergence was determined when the root-mean-squared (RMS) deviation between the normalized forward propagated intensity and the measured intensity (ground truth) at each plane is below a certain threshold. Unlike the technique reported in Ref. (52), we used Kirchhoff–Fresnel and Fraunhofer diffraction formulas to propagate the field in the forward and backward directions.

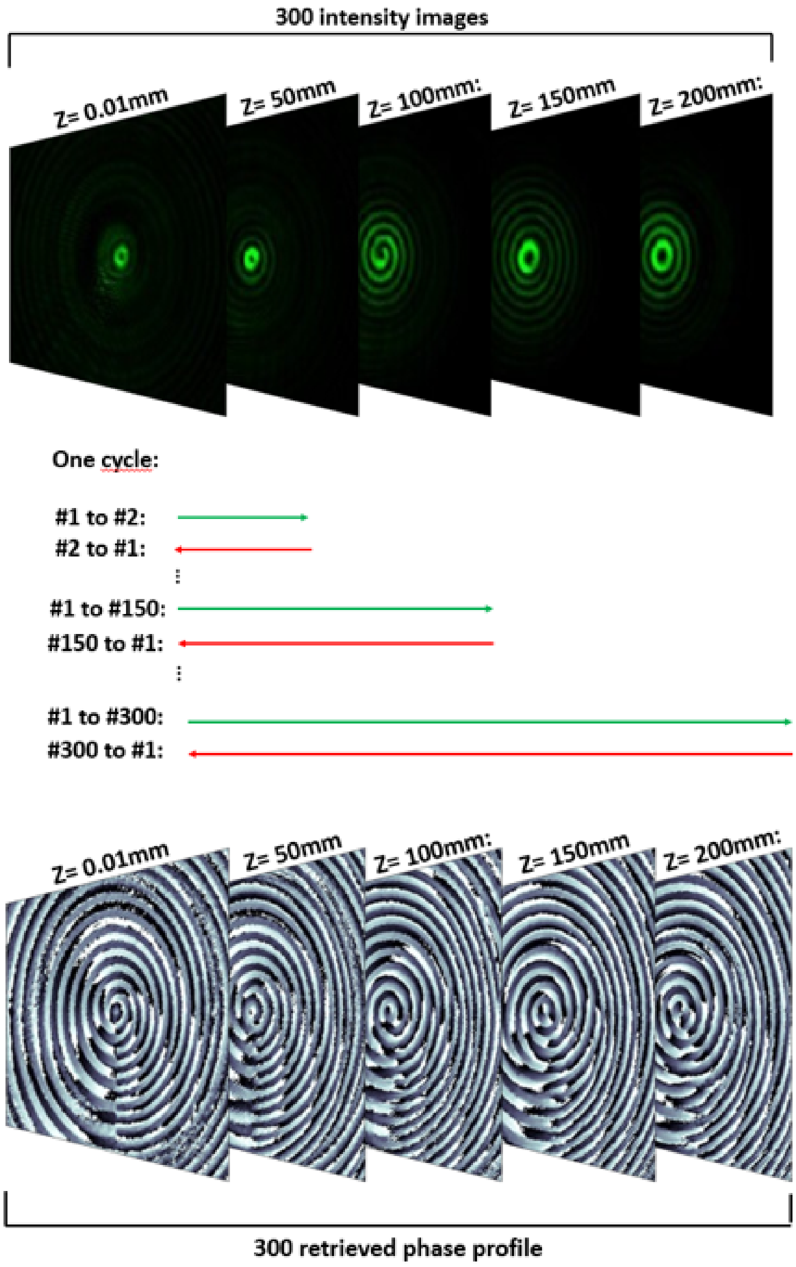

Figure S8: Schematic diagram illustrating our iterative single-beam multiple intensity reconstruction phase retrieval algorithm. From a set of 2D intensity measurements with equal separation along the optical path (top). The corresponding 2D phase map (bottom) can be fully retrieved by following via iterative calculation.

9 Manifestation of Logarithmic Spirals in Nature

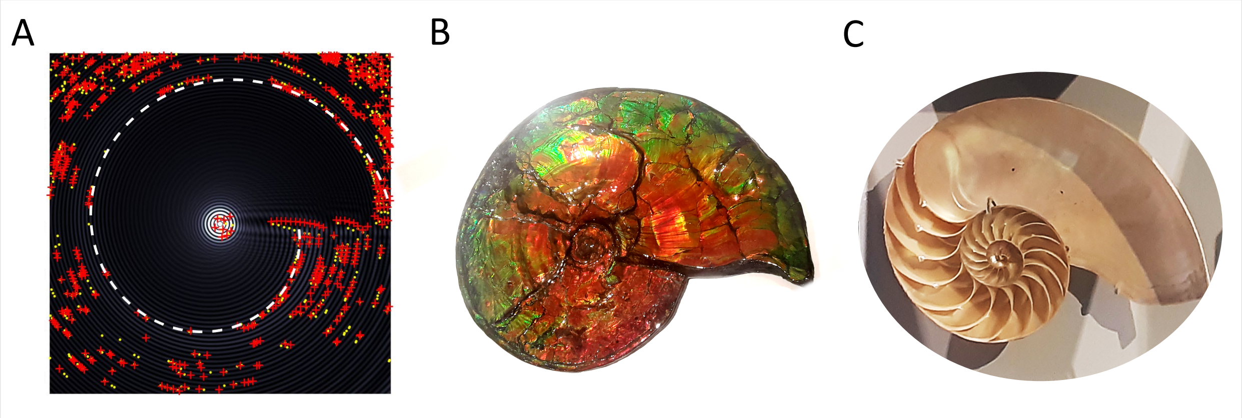

Figure S9: Rotatum and logarithmic spirals. **(A)** Simulated 2D intensity profile of a vortex beam with optical rotatum at a propagation distance of $z = 1.5$ cm. Here, the vortex is composed of 91 Bessel vortex beams ($N = 45$). The red and yellow markers denote the positive and negative phase singularities. The contour of these singularities follows a logarithmic spiral pattern that resembles many phenomena in nature such as pattern formation in crystals and seashells. The white curve denotes a a fitted logarithmic spiral function with the following parameters $r = ae^{k\phi}$; $a = 165$, $k = 0.12$ and $\phi = [0, 2\pi]$ plotted on the square grid of 665 by 665 unit pixels. **(B)** An image of Aragonite which is a carbonate mineral and one of the three most common naturally occurring crystal forms of calcium carbonate. Aragonite is formed by biological and physical processes, including precipitation from marine and freshwater environments. **(C)** The chambered nautilus, also called the pearly nautilus, is the best-known species of nautilus. The shell, when cut away, reveals a lining of lustrous nacre and displays a nearly perfect equiangular spiral.

Supplementary Video 1: Evolution of intensity and phase profiles of vortex beams with linearly growing orbital angular momentum along the optical path, exhibiting spatial self-torque.

Supplementary Video 2: Evolution of vortex beams whose orbital angular momentum experiences linear growth then decay along the optical path.

Supplementary Video 3: Evolution of vortex beams whose orbital angular momentum decays then grows following a linear dependence along the optical path.

Supplementary Video 4: Evolution of vortex beams whose orbital angular momentum experiences quadratic chirp along the optical path, exhibiting optical rotatum.
